# Balloon-occluded retrograde transvenous obliteration for gastric varices: the relationship between the clinical outcome and gastrorenal shunt occlusion

**DOI:** 10.1186/1471-2342-10-2

**Published:** 2010-01-14

**Authors:** Kenichi Katoh, Miyuki Sone, Atsuo Hirose, Yoshihiro Inoue, Yasuhisa Fujino, Makoto Onodera

**Affiliations:** 1 Department of Radiology, Iwate Medical University, Morioka, Japan; 2 Department of Radiology, Morioka Red Cross Hospital, Morioka, Japan; 3 Department of Critical Care Medicine, Iwate Medical University, Morioka, Japan

## Abstract

**Background:**

The rupture of gastric varices is associated with high mortality rate. Balloon-occluded retrograde transvenous obliteration (B-RTO), a minimally invasive procedure that was introduced in the mid-1990s, has been widely accepted in Japan. Several reports have indicated that B-RTO yields satisfactory results; however, few reports have discussed the recurrence of gastric varices after this therapy. The purpose of this study is to retrospectively evaluate the technical aspects of B-RTO and the recurrence of gastric varices after treatment with this procedure.

**Methods:**

B-RTO was performed in 47 patients with gastric varices, who were at a risk of variceal ruptures and who may or may not have had a history of variceal bleeding. We injected a sclerosing agent into the gastric varices for 30-60 minutes. To evaluate the therapeutic efficacy of the technique, we obtained contrast-enhanced computed tomography (CT) scans 5 days after B-RTO. As a general rule, if the gastric varices did not appear thrombosed, we repeated the procedure 7 days after the first procedure.

**Results:**

B-RTO was a technical success in 37 patients. It was performed once in 26 patients, twice in 6 patients, thrice in 2 patients, and 4 times in 3 patients. Contrast-enhanced CT scans obtained after B-RTO showed thrombosed gastrorenal shunts in 29 patients and patent gastrorenal shunts in 8 patients. The gastric varices recurred in 2 patients who had patent gastrorenal shunts. The overall cumulative relapse-free rate of gastric varices was 90% at 5 years after B-RTO.

**Conclusions:**

B-RTO is an effective treatment modality for gastric varices. Moreover, obliteration of the gastrorenal shunt as well as the gastric varices appears to be important for the treatment of gastric varices.

## Background

The rupture of gastric varices is associated with a mortality rate of 25-55% because it leads to extensive blood loss as compared to the blood loss because of the rupture of esophageal varices [1-4]. Because of poor liver function and rapid blood flow in patients with gastric varices, the development of effective treatment for this condition is a challenge. Gastric varices can be treated by endoscopic injection therapy with cyanoacrylate, but there is a risk of migration of this compound into systemic circulation through the inferior vena cava via the gastrorenal shunt [5]. Balloon-occluded retrograde transvenous obliteration (B-RTO), a minimally invasive procedure that was introduced in the mid-1990s, has been widely accepted in Japan. In the standard technique, gastric varices are thrombosed using a sclerosing agent that is injected through a balloon catheter. Several reports have indicated that B-RTO yields satisfactory results [6-11]; however, few reports have discussed the recurrence of gastric varices after this therapy. The present study was conducted to evaluate the clinical outcomes of B-RTO performed for the treatment of gastric varices and to investigate the recurrence of these varices.

## Methods

### Patients

Between July 1999 and September 2008, 47 patients (30 men and 17 women; age range, 45-79 years; mean age, 61 years) who had gastric varices with gastrorenal or gastrocaval shunts underwent B-RTO at our hospital. The main clinical characteristics of these 47 patients are presented in Table 1. The underlying liver disease was viral hepatitis-related liver cirrhosis in 28 patients, alcoholic liver cirrhosis in 17 patients, and other conditions in 2 patients. According to the Child-Pugh classification system, 28 patients were categorized into class A, 18 into class B, and 1 into class C. The gastric varices were subdivided into 2 types--gastroesophageal varix (GOV) and isolated gastric varix (IGV)--according to the method established by Sarin [2,3]. GOVs extend beyond the gastroesophageal junction and are always associated with esophageal varices. They are further subdivided into GOV-1 and GOV-2: GOV-1 appears as a continuation of esophageal varices and extend for 2-5 cm below the gastroesophageal junction along the lesser curvature of the stomach, and GOV-2 extends beyond the gastroesophageal junction towards the fundus of the stomach. IGVs are gastric varices that are not accompanied by esophageal varices; they are subdivided into IGV-1 and IGV-2. IGV-1 is located in the fundus of the stomach, and IGV-2 is isolated ectopic varix which can be found in all regions of the stomach. According to this classification, GOV-1s were found in 8 patients (17%), GOV-2s in 20 patients (43%), and IGV-1s in 19 patients (40%). Of these, 7 patients had previously had episodes of bleeding from gastric varices; to control the bleeding, endoscopic injection therapy with cyanoacrylate had been administered as an emergency procedure to 3 patients, and endoscopic variceal ligation had been performed in another 3. However, some varices persisted or bleeding recurred. Therefore, these patients were scheduled for B-RTO. In 1 of the patients, gastric variceal bleeding ceased during endoscopy, and B-RTO was scheduled without any endoscopic therapy. In the remaining 40 patients, the procedure was performed as a prophylactic treatment. The risks of rupture, such as progressive varices and red spots on gastric varices, were identified by endoscopy. The attending physician attempted to use H2 blockers to prevent gastric ulcer or beta-blockers to reduce portal vein pressure in some patients before the B-RTO procedure was performed. The institutional review board permitted the data collection and approved the study. Informed written consent was obtained from all the patients, and the research was carried out in accordance with the Helsinki Declaration.

**Table 1:** Main characteristics of the 47 patients

Sex (M/F)	30/17
Age (mean)	45 - 79 (61)
Cause of liver disease, no. (%)	
Postviral	28 (60)
Alcoholic	17 (36)
Autoimmune hepatitis	1 (1)
Extra hepatic portal vein stenosis post surgery	1 (1)
Child-Pugh class, no. (%)	
A	28 (60)
B	18 (38)
C	1 (2)
Previous gastic variceal bleeding, no. (%)	
Present	7 (15)
Absent	40 (85)
Sarin's classification	
GOV-1	8 (17)
GOV-2	20 (43)
IGV-1	19 (40)

### Procedure

**Figure 1:**
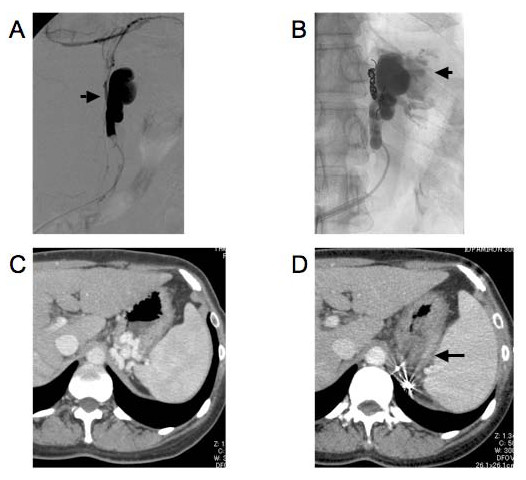
**Balloon-occluded retrograde transvenous obliteration (B-RTO) performed on a 47-year-old woman with hepatitis C virus-related liver cirrhosis**. A. A balloon catheter was inserted into the gastrorenal shunt. The gastric varices could not be clearly visualized on the venogram because the contrast medium had leaked into the retroperitoneal collateral veins (arrow). B. The retroperitoneal collateral veins were catheterized using a microcatheter and embolized using metallic coils. The gastric varices (arrow) could be clearly visualized on a venogram obtained after embolization of the retroperitoneal veins. C. A contrast-enhanced CT scan obtained before the B-RTO procedure showed gastric varices and gastrorenal shunts. D. A contrast-enhanced CT scan obtained 5 days after the first B-RTO procedure showed complete thrombosis of the gastric varices (arrowhead).

The B-RTO procedure is illustrated in Fig. 1. Contrast-enhanced computed tomography (CT) was performed before the procedure in order to visualize the anatomy of the gastric varices and confirm the presence of a gastrorenal shunt. An 8-French (Fr) hook-shaped sheath (Clinical Supply, Gifu, Japan) was inserted into the left renal vein via the right femoral vein. If the gastric varices did not drain via a gastrorenal shunt, the gastrocaval shunt was directly catheterized. Depending on the size of the shunt, a 5-Fr balloon catheter (balloon diameter, 11 mm; Clinical Supply, Gifu, Japan) or a 6-Fr balloon catheter (balloon diameter, 20 mm; Clinical Supply, Gifu, Japan) was inserted. When the size of the shunt was extremely large, a 7-Fr balloon catheter (balloon diameter, 30 mm; Nipro, Osaka, Japan) was also used. Initially, balloon-occlusion venography was performed with an inflated balloon; however, the gastric varices did not appear opacified because the collateral veins, such as the inferior phrenic vein or the pericardiacophrenic vein, were dilated. These collateral veins were embolized using metallic coils. In case the collateral veins were small and numerous, we used a small amount of an ethanolamine oleate-iopamidol (EOI) mixture containing 10% ethanolamine oleate (Oldamin; Takeda Pharmaceuticals, Osaka, Japan) diluted with an equal amount of iopamidol (Iopamiron 300; Bayer Healthcare, Osaka, Japan). After the occlusion of the collateral veins, balloon-occlusion venography was performed again to confirm that the contrast medium had filled the gastric varices. Even if the gastric varices were not sufficiently filled by the contrast medium after the occlusion of the collateral veins, the splenic artery was temporarily occluded by a balloon catheter in order to reduce the inflow route from the short gastric vein or posterior gastric vein. We injected the EOI mixture into the shunt and gastric varices for 30-60 minutes without prolonged occlusion with a balloon catheter. During the injection, we monitored the position of the balloon catheter by using fluoroscopy to avoid catheter dislodgement, and we subsequently withdrew as much of the agent as possible. In order to prevent renal damage due to EOI-induced hemolysis, 4000 units of haptoglobin (Yoshitomi, Osaka, Japan) was intravenously administered before the EOI infusion. To evaluate the therapeutic efficacy of gastric varices, we performed contrast-enhanced CT 5 days after B-RTO. As a general rule, if the gastric varices were not thrombosed, B-RTO was repeated 7 days after the first procedure. Further, if the varices were not thrombosed even after the second or third procedure, a repeat B-RTO procedure was scheduled after 2 or 3 months.

### Follow-up examination

Follow-up CT and endoscopy were performed every 3-6 months. Depending on the patient's condition, the attending physician decided the follow-up interval. The recurrence of gastric varices was evaluated by performing follow-up endoscopy or contrast-enhanced CT. Follow-up evaluation of the esophageal varices was performed via endoscopy. When the follow-up endoscopy revealed newly developed esophageal varices, red spots on preexisting esophageal varices, or esophageal variceal bleeding, the esophageal varices were regarded as having worsened.

### Definitions and Statistical Analysis

When the sclerosing agent was successfully injected and complete thrombosis of the varices, as observed on a contrast-enhanced CT scan after the first or subsequent B-RTO procedure, was achieved, B-RTO was considered successful. B-RTO failure was classified as either technical failure or thrombosis failure. Technical failure was defined as the inability of performing the B-RTO procedure. Thrombosis failure was defined as technically successful completion of the B-RTO procedure without thrombosis of gastric varices on the follow-up contrast-enhanced CT scan. The rates of patient survival and recurrence of the gastric and esophageal varices were calculated using the Kaplan-Meier method.

## Results

### Outcome of B-RTO

In 37 of the 47 patients (79%), B-RTO was technically successful and complete thrombosis of the varices was observed on the contrast-enhanced CT scans. Of these 37 patients, B-RTO was performed once in 26 patients (70%), twice in 6 patients (16%), thrice in 2 patients (6%), and 4 times in 3 patients (8%). However, this technique was not successful in the other 10 patients. In 6 of these 10 patients, B-RTO could not be performed (technical failure) for the following reasons: (1) the gastrorenal shunt could not be occluded with the balloon catheter because the shunt was extremely large and because of rapid blood flow in the area (2 cases); (2) catheterization was difficult owing to the presence of fine and tortuous gastrocaval or gastrorenal shunts (2 cases); and (3) the gastric varices could not be visualized owing to the presence of many retroperitoneal veins (2 cases). Thrombosis failure was observed in 4 patients. In this group, B-RTO was performed once in 2 patients, thrice in 1 patient, and 4 times in 1 patient, but thrombosis of the varices could not be achieved. Among the 10 patients in whom this procedure failed, 3 underwent endoscopic injection therapy instead of B-RTO. The other patients did not provide their consent for further treatment.

### Complications

Complications occurred during the procedures in the case of 4 patients. In 1 patient, iatrogenic injury to the gastrorenal shunt occurred during catheterization. The patient's vital signs remained normal, but no additional angiographic analyses or intervention was carried out at that time. After 2 months, B-RTO was attempted for the second time, and it was successful. In another patient, a microcoil (diameter, 3 mm) that was used for embolization of the inferior phrenic vein migrated to the distal region of the right pulmonary artery. Since the coil was small and since its potential adverse effects were considered to be minimal, we did not attempt to retrieve it. In the other 2 patients, the sclerosing agent entered systemic circulation within 5 minutes owing to dislodgement of the balloon catheter. One of these patients experienced chest discomfort, and his pulse oxygen saturation (SpO_2_) levels transiently decreased. Hence, no additional intervention was performed at that time, but B-RTO was repeated at a later date.

### Survival

**Figure 2:**
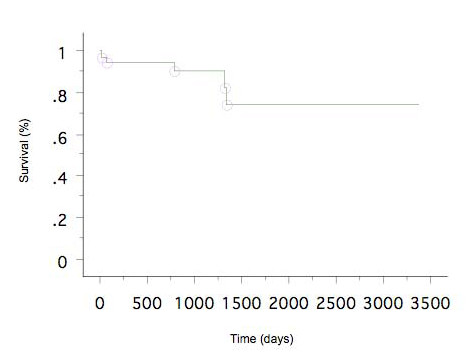
**Cumulative survival rate after B-RTO**.

Among the 37 patients who successfully underwent B-RTO, 3 were lost to follow-up. Thus, 34 patients were followed up and their cases reviewed. The median follow-up period was 1147 days (range, 15-3375 days), during which time 5 patients died. Of these, 2 died soon after the procedure: 1 patient who underwent endoscopic injection sclerotherapy for esophageal varices after B-RTO died after 15 days of sepsis caused by severe diabetes mellitus; the other died after 71 days because of rupture of esophageal varices. Of the other 3 patients who died, 2 died from hepatocellular carcinoma (784 days and 1326 days after B-RTO) and 1 died of hepatic failure (1345 days after B-RTO). The overall cumulative survival rate at 1, 3, and 5 years after B-RTO was 92%, 90%, and 73%, respectively, as determined by the Kaplan-Meier method (Fig. 2).

### Recurrence of gastric varices and gastrorenal shunt patency

**Figure 3:**
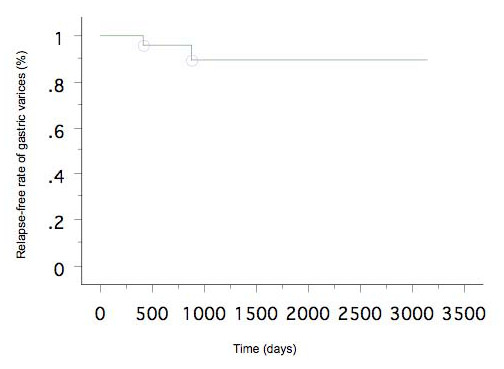
**Cumulative relapse-free rate of gastric varices after B-RTO**.

**Figure 4:**
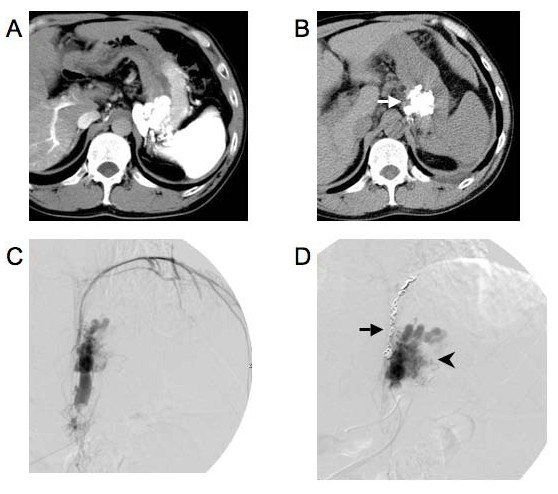
**B-RTO performed on a 49-year-old man with alcohol-related liver cirrhosis**. A CT scan obtained during arterioportography showed gastric fundal varices. B. Bleeding of the gastric varices occurred before the scheduled B-RTO procedure, and endoscopic injection sclerotherapy (EIS) using cyanoacrylate was performed as an emergency procedure to control the bleeding. A plain CT scan obtained after EIS showed that the sclerosing agent was almost uniformly distributed throughout the network of gastric varices (arrow), except in the gastrorenal shunt. The scheduled B-RTO was cancelled because the bleeding of the gastric varices ceased after the emergency EIS. C. The patient had a history of repeated gastric variceal bleeding and was referred to our department for a B-RTO procedure 4 years after he had received EIS for the first time. The venogram revealed a gastrorenal shunt; however, the gastric varices could not be clearly visualized because the contrast medium had leaked into systemic circulation. D. A retrograde venogram obtained after coil embolization of the inferior phrenic vein (arrow) clearly showed the recurrence of gastric varices (arrowhead). The gastric varices were completely thrombosed after the first B-RTO procedure, as determined by CT during follow-up.

As mentioned above, among the 37 patients who successfully underwent B-RTO, 3 were lost to follow-up and 2 died soon after the procedure. The remaining 32 patients were followed up for detecting the recurrence of gastric varices and progression of esophageal varices. During the median follow-up period of 746 days (range, 134-3136 days), the gastric varices recurred in 2 patients (after 13 months in one and after 28 months in the other). The overall cumulative relapse-free rate of gastric varices was 90% at 3 and 5 years after the first B-RTO (Fig. 3). We also analyzed perigastric gastrorenal shunt thrombosis as well as gastric variceal thrombosis on contrast-enhanced CT scans obtained after the B-RTO procedure. In the 32 patients who were followed up, gastrorenal shunt thrombosis, as well as gastric variceal thrombosis, was observed in 25 patients, and gastric variceal thrombosis without gastrorenal shunt thrombosis was observed in 7 patients. Gastric variceal recurrence occurred in 2 cases of gastric variceal thrombosis without gastrorenal shunt thrombosis. These 2 patients underwent additional B-RTO procedures, and complete thrombosis of the gastric varices and gastrorenal shunt were achieved. We also treated 3 patients in whom the gastric varices recurred after the emergency endoscopic injection therapy using cyanoacrylate. In these patients, the gastrorenal shunt remained patent, and the residual gastric varices appeared opacified during balloon-occluded retrograde venography; B-RTO was successful for these patients (Fig. 4). However, in one of these cases, contrast-enhanced CT revealed a patent gastrorenal shunt, and the gastric varices recurred 28 months after B-RTO (as mentioned above).

### Progression of esophageal varices

**Figure 5:**
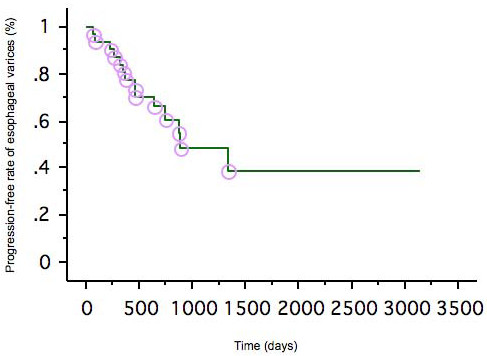
**Cumulative progression-free rate of esophageal varices after B-RTO**.

In contrast, esophageal varices worsened in 14 patients during the follow-up period (median, 656 days; range, 80-3136 days). The overall cumulative progression-free rates of esophageal varices at 1, 3, and 5 years were 93%, 47%, and 38%, respectively (Fig. 5). Among these 14 patients, 2 patients had bleeding esophageal varices and 7 patients were at a risk of bleeding from esophageal varices. They underwent endoscopic injection sclerotherapy.

## Discussion

The risk of bleeding from gastric varices is lower than that of bleeding from esophageal varices, but rupture of gastric varices is a serious condition associated with a mortality rate of 25-55% [1-4]. The risk factors for hemorrhage from gastric varices are large-sized varices, the presence of red spots on varices, and a severe Child's status [4]. The available treatment options for gastric varices include shunt surgery, endoscopic injection sclerotherapy (EIS) with cyanoacrylate or ethanolamine oleate, and transjugular intrahepatic portosystemic shunting (TIPS). Gastric varices frequently develop from the short and posterior gastric veins; this is in contrast to esophageal varices, which are known to develop mainly from the coronary vein [12]. In gastric varices with a large gastro-renal shunt, a sclerosing agent could even enter the renal shunt and from there, the inferior vena cava. Therefore, prophylactic EIS is not recommended for the treatment of gastric varices. B-RTO, which was introduced in the mid-1990s, has proven to be an effective method for this purpose. Since then, it has been used for the treatment of hemorrhagic gastric varices and for prophylactic treatment in Japan. Ninoi et al. reported that transcatheter sclerotherapy procedures such as B-RTO may be more effective than TIPS in controlling gastric variceal bleeding [13]. In our study, the gastric varices recurred in only 2 patients, and no bleeding was observed. The overall cumulative relapse-free rate was 90% at 3 and 5 years after B-RTO. However, worsening of esophageal varices is one problem after B-RTO in long-term follow up as widely reported in some articles [8,10,11]. In our study, 44% of patients experienced worsening of their esophageal varices during median follow-up period of 656 days. Therefore, endoscopic examination is extremely important for discovering worsening of esophageal varices after B-RTO.

Although B-RTO is more efficient than other techniques and procedures and provides better long-term results, it may be difficult to achieve technical success in cases where the collateral vessels are numerous and large. Even in cases where the collateral vessels are occluded, multiple B-RTO procedures are frequently required to completely obliterate the gastric varices. Moreover, alternative procedures such as percutaneous transhepatic sclerotherapy may be necessary in some cases where B-RTO is unsuccessful [13].

**Figure 6:**
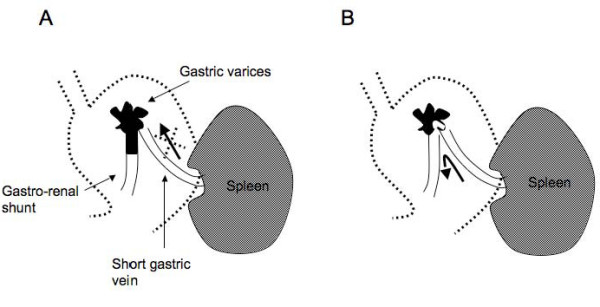
**Illustration of gastric varices after B-RTO**. A. The gastric varices and gastrorenal shunt are thrombosed. B. The gastric varices are thrombosed, but a small region of the varices remains and the gastrorenal shunt are patent. These conditions may induce gradual regrowth of the varices.

In our study, 32 patients who successfully underwent B-RTO were followed-up. Contrast-enhanced CT performed after the B-RTO procedure revealed thrombosis of the gastrorenal shunt as well as gastric varices in the case of 25 patients, and thrombosis of the gastric varices with patency of the gastrorenal shunts in the case of 7 patients. The gastric varices recurred in 2 patients who had patent gastrorenal shunts--at 13 months after B-RTO in one and at 28 months after B-RTO in the other. In addition, the varices recurred after endoscopic injection therapy using cyanoacrylate in the case of 3 patients in whom the gastrorenal shunts remained patent. The B-RTO procedure is performed to achieve retrograde obliteration of gastric varices through a draining vein. Even in cases where the gastric varices appear thrombosed on contrast-enhanced CT scans, a small region of the varices and the gastrorenal shunt may remain patent and induce regrowth of the gastric varices (Fig. 6). Several investigators have reported that the use of a microcatheter during B-RTO enables selective obliteration of gastric varices while maintaining the patency of the gastrorenal shunt [14,15]. However, as observed in our study, a patent gastrorenal shunt may cause recurrence of gastric varices. Therefore, obliteration of the gastrorenal shunt as well as the gastric varices is essential.

Recently, a group of Korean investigators also reported the use of B-RTO for the treatment of gastric varices [16]. They reported that B-RTO is an effective treatment for the obliteration of gastric varices. However, postprocedural liver failure resulted in the death or the discharge as hopeless within 2 months of 6 patients, and procedure-related death occurred in the case of 2 patients who were classified as C or late B (scores of 8 or 9) according to the Child-Pugh classification system. Therefore, they concluded that the use of this procedure in severely compromised patients should be considered carefully. In contrast to their results, we did not experience procedure-related liver failure; this was probably because the majority of our patients had early-stage liver disease. B-RTO is highly efficacious for the treatment of gastric varices; therefore, in Japan, it is often used as a prophylactic treatment technique before the rupture of gastric varices. Some authors have insisted that prophylactic treatment results in good survival rates [17]. B-RTO is widely accepted in Japan, but not in other parts of the world. In particular, there is no mention of B-RTO in the AASLD (American Association for the Study of Liver Diseases) guidelines [18]. For this therapy to be accepted universally, randomized controlled trials are required. In addition, further studies are required to evaluate the indications of this therapy in patients with poor liver function and the necessity of prophylactic treatment.

## Conclusion

In summary, B-RTO is an effective method for the treatment of patients with gastric varices. Moreover, obliteration of the gastrorenal shunt as well as the varices appears to be particularly important for preventing the recurrence of gastric varices in this procedure.

## Competing interests

The authors declare that they have no competing interests.

## Authors' contributions

All authors have made substantial contributions to in the design of this article. KK has contributed to conception and design of the study as well as the final revision. KK is the principal interventional radiologist of many cases. MS and AH have contributed to conception, design, interpretation of data and discussion as well as in interventional radiological technique. YI, YF and MO have contributed to conception, design, interpretation of data and discussion. All authors have read and approved the final manuscript.

## Pre-publication history

The pre-publication history for this paper can be accessed here:

http://www.biomedcentral.com/1471-2342/10/2/prepub
